# Involvement of NLRP3 Inflammasome in Methamphetamine Augmentation of SARS-CoV-2 N-Protein-Induced Neuroinflammation in Rat Microglial Cells

**DOI:** 10.3390/ijms27156960

**Published:** 2026-08-03

**Authors:** Debashis Dutta, Jianuo Liu, Huangui Xiong

**Affiliations:** Department of Pharmacology and Experimental Neuroscience, College of Medicine, University of Nebraska Medical Center, Omaha, NE 68198-5880, USA

**Keywords:** SARS-CoV-2, N-protein, long COVID, methamphetamine (meth), microglia, NLRP3 inflammasome, cytokine storm, neuroinflammation

## Abstract

Severe acute respiratory syndrome coronavirus 2 (SARS-CoV-2) infection causes an immune-mediated neurological syndrome, which persists long after infection. Mechanisms for SARS-CoV-2-associated neurological complications are multifactorial, with an increased risk of drug abuse such as methamphetamine (meth). SARS-CoV-2 infection and its viral proteins play pivotal roles in coronavirus disease 2019 (COVID-19)-associated neuroinflammation, which can lead to long COVID. We hypothesize that meth augments activation of the microglial NOD-, leucine-rich repeat, and pyrin domain-containing protein 3 (NLRP3) inflammasome by the SARS-CoV-2 nucleocapsid (N) protein, resulting in neuroinflammation. To test this hypothesis, we investigated the effect of N-protein and meth on NLRP3 inflammasome activation in primary rat microglial cultures using enzyme-linked immunosorbent assay (ELISA), Reverse Transcription quantitative Polymerase Chain reaction (RT-qPCR), western blot (WB), and immunofluorescence assay (IFA). Our results showed that meth augmented N-protein-induced microglial activation, as evidenced by increased ionized calcium-binding adapter 1 (Iba-1) expression. The addition of meth to the microglial cultures treated with N-protein increased proinflammatory cytokine production. Meth augmentation of N-protein-induced neuroinflammation was further supported by increased inducible nitric oxide synthase (iNOS)-mediated nitric oxide (NO) production. The effects of meth on N-protein-associated inflammatory responses were significantly attenuated by MCC950, a specific NLRP3 inhibitor. Moreover, meth-associated NLRP3 activation was either blocked by the opioid sigma1-receptor (σ1_-_R) inhibitor BD1047 or by σ1R siRNA knockdown. Taken together, these results demonstrated that meth augmented SARS-CoV-2 N-protein-induced neuroinflammation via microglial σ1_-_R and the NLRP3 inflammasome, which may underlie the pathogenesis of neurological manifestations in COVID-19, such as long COVID with meth abuse. These results may also underscore the impact of drug abuse on long COVID and provide targets for the development of therapeutic strategies to control the neurological outcomes of long COVID.

## 1. Introduction

The causative agent of the coronavirus disease 2019 (COVID-19) pandemic is severe acute respiratory syndrome coronavirus 2 (SARS-CoV-2), which poses an imminent threat to global public health [1,2,3]. SARS-CoV-2 is phylogenetically related to previous coronavirus outbreaks, such as severe acute respiratory syndrome coronavirus (SARS-CoV) and Middle East Respiratory Syndrome (MERS) [4,5]. The emergence of SARS-CoV-2 was first reported in Wuhan, China [3,6,7,8]. The virus primarily infects the human respiratory tract, causing symptoms such as fever, dry cough, fatigue, shortness of breath, body aches, and headache [9,10,11]. Although the disease is mild in most cases, in a small number of infected individuals, it can worsen and progress to acute respiratory distress syndrome (ARDS), coagulation dysfunction, and death [12,13,14]. It is generally believed that SARS-CoV-2 infection triggers severe inflammatory responses and that dysregulated innate immunity underlies the severity of COVID-19 [15,16,17,18]. Approximately 36% of SARS-CoV-2-infected individuals develop neurological symptoms, of which 25% are attributed to direct involvement of the central nervous system (CNS) [19]. These symptoms include dizziness, headache, impaired consciousness, and seizures [20]. There is a rise in neurological symptoms post-COVID-19, referred to as post-acute sequelae of COVID-19 (PASC) or long COVID, with an estimated worldwide prevalence of 30.8% [21,22,23,24,25,26,27,28]. These neurological manifestations are associated with immune dysregulation, dysregulated cytokines, neurovascular injury, and CNS inflammation.

It has been demonstrated that SARS-CoV-2 can induce cytokine secretion by activating NOD, leucine-rich repeat, and pyrin domain-containing protein 3 (NLRP3) inflammasomes, which is associated with the severity of COVID-19 [18,19,29,30,31]. To date, SARS-CoV-2-mediated NLRP3 inflammasome activation has been reported in human monocytes, macrophages, and lung cells, all of which are implicated in cytokine storms [30,32]. SARS-CoV-2 nonstructural protein ORF3a (Viroprin) [33,34] and structural proteins, including the spike protein (S) [35,36] and nucleocapsid (N) [37], can also trigger NLRP3 inflammasome activation in the cells mentioned above. Nevertheless, NLRP3 inflammasome activation and the resulting release of interleukin-1beta (IL-1β) and other cytokines can trigger cytokine storms and ARDS [38,39,40]. Several studies have reported that the SARS-CoV-2 S-protein can activate the NLRP3 inflammasome in microglial cells [41,42,43], and that NLRP3 activation induces neurotoxic astrocytes [44]. Thus, studies to date indicate that SARS-CoV-2 and its viral proteins can activate microglial cells via the NLRP3 inflammasome, potentially leading to neurological complications. A recent study reported that the severity of long COVID is directly associated with NLRP3 activation and the inflammatory response during acute SARS-CoV-2 infection [45].

Given their key roles in maintaining CNS homeostasis, regulating CNS function, and mediating neurological outcomes of various perturbations, microglia are substantially involved in long COVID. Microglia are a resident macrophage population in the CNS, and their activation is observed in various neurodegenerative diseases. Research suggests that microglial NLRP3 activation is closely linked to neurodegeneration [46]. When microglial NLRP3 is excessively activated by SARS-CoV-2, this may trigger a cascade of inflammatory responses that release cytokines, which can directly damage neurons and contribute to the progression of PASC/long COVID. Indeed, a post-mortem study of three SARS-CoV-2-infected cases revealed the involvement of the microglial NLRP3 inflammasome in the cerebral pathogenicity of COVID-19 [47,48]. This demonstrates the activation of the NLRP3 inflammasome; NLRP3 inflammasome-mediated proinflammatory cytokine secretion can lead to a neuroinflammatory environment and consequences in neuro-COVID, long COVID, and/or PASC [49].

The COVID-19 pandemic imposed societal constraints, including social distancing and lockdowns. These constraints have affected people’s psychological well-being [50]. In addition, substance abuse reportedly increased during the COVID-19 pandemic, specifically among vulnerable groups [51,52]. This rise has taken a greater toll on people during the COVID-19 pandemic and beyond. Increased drug abuse, especially of methamphetamine (meth), could worsen the neuroimmune milieu by altering cytokine signaling mediated by NLRP3 inflammasomes [53,54,55], resulting in enhanced SARS-CoV-2 infection, pathogenesis, and disease outcomes [56]. Therefore, exploring the mechanisms underlying SARS-CoV-2- and meth-induced neuroinflammation and their correlations with the pathogenesis of PASC (long COVID) is critical for developing therapeutic strategies.

In the present study, we demonstrated that the SARS-CoV-2 N-protein activated NLRP3 inflammasomes in primary rat microglial cultures, resulting in IL-1β maturation and release, as well as increased levels of other proinflammatory cytokines, including Tumor Necrosis Factor Alpha (TNF-α), Interleukin-6 (IL-6), and Interleukin-18 (IL-18). The N-protein-mediated increase in proinflammatory cytokines and neurotoxic nitric oxide (NO) in microglia can trigger a cytokine storm, leading to neuroinflammation and neuronal damage. Furthermore, the SARS-CoV-2 N-protein-mediated activation of the microglial NLRP3 inflammasome and the resulting outcomes were augmented by meth. The meth augmentation of N-protein-induced NLRP3 activation can be blocked or attenuated by MCC950, an NLRP3 inflammasome-specific inhibitor; by BD1047, an opioid sigma-1 receptor (σ1-R) blocker; or by genetic knockdown of σ1-R, demonstrating that meth augmentation of SARS-CoV-2 N-protein-mediated NLRP3 inflammasome activation occurs via σ1-R in rat microglial cultures.

## 2. Results

### 2.1. Meth Treatment Enhances N-Protein-Mediated Microglial Activation

**Figure 1:**
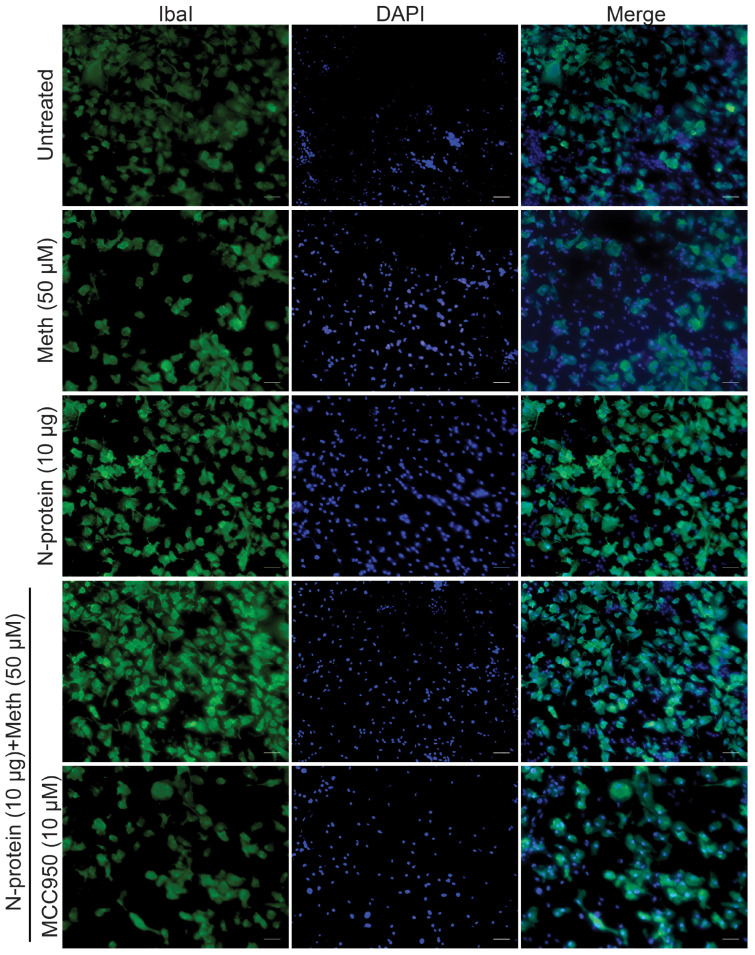
Effect of meth on SARS-CoV-2 N-protein-induced microglial activation. Microglial activation induced by the SARS-CoV-2 N-protein and meth was evaluated by fluorescence microscopy. PFA-fixed microglial cells stained with the microglia-specific marker Iba1 showed microglial activation upon N-protein treatment, which was enhanced upon meth treatment in N-protein-treated cells. Microglial cultures were pretreated with 10 µM MCC950 two hours before N-protein treatment. Scale bar = 100 µm.

SARS-CoV-2 and its viral proteins are known to induce microglial activation in humans and animal models [41,57,58,59,60]. We observed microglial activation induced by the SARS-CoV-2 N-protein using immunofluorescence (IF) staining for IbaI. We found increased IbaI expression in microglial cultures after N-protein treatment (Figure 1). Meth treatment further enhanced microglial IbaI expression in N-protein-treated cells. Pretreatment of microglial cells with the NLRP3-specific inhibitor MCC950 blocked microglial activation, suggesting that meth augmentation of SARS-CoV-2 N-protein-mediated microglial activation is via NLRP3 inflammasome activation. Thus, the observed N-protein-induced and meth-enhanced microglial activation may contribute to the CNS proinflammatory environment. Thus, with this, we can assume that this N-protein-induced and meth-enhanced microglial-activation-mediated proinflammatory milieu could lead to persistent neuroinflammation that may contribute to PASC or long COVID exacerbation in people who abuse drugs such as meth.

### 2.2. Meth Augments N-Protein-Induced Cytokine Production and Caspase-1 Activity

In addition to primarily respiratory complications, SARS-CoV-2 infection can spread to multiple organs, including the CNS, causing multiorgan complications and neurological manifestations. COVID-19-mediated complications are partly due to excessive cytokine production (cytokine storm), which occurs via activation of the NLRP3 inflammasome. The exact mechanism by which SARS-CoV-2 enters the CNS and causes infection remains debated. However, viral proteins have been detected in post-mortem brain tissue, and the NLRP3 inflammasome has been implicated in cerebral pathogenicity [47,48]. In this study, we simulate viral protein-mediated complications by treating with the SARS-CoV-2 N-protein. Treatment of rat neonatal microglial cultures with increasing concentrations of N-protein resulted in a dose-responsive increase in IL-1β secretion (Figure S1). We measured the neurotoxic and proinflammatory cytokines released by microglial cultures treated with the SARS-CoV-2 N-protein, which mediate N-protein-induced neuroinflammation. Following N-protein treatment, we measured IL-1β, IL-6, TNF-α, and IL-18 (Figure S1A–D) and found increases in these cytokines with increasing treatment concentration. Pretreatment of microglial cells with the NLRP3-specific inhibitor MCC950 did not increase cytokine production after N-protein treatment, indicating that NLRP3 inflammasome activation mediates the enhancement of cytokine secretion. For experimental control, we included lipopolysaccharide (LPS) at concentrations of 100 and 500 ng/mL to activate the inflammasome. Furthermore, treatment with N-protein increased caspase-1 activity in microglial culture supernatants (Figure S1E). Pretreatment of microglial cells with the NLRP3-specific inhibitor MCC950 did not increase caspase-1 activity (Figure S1E).

**Figure 2:**
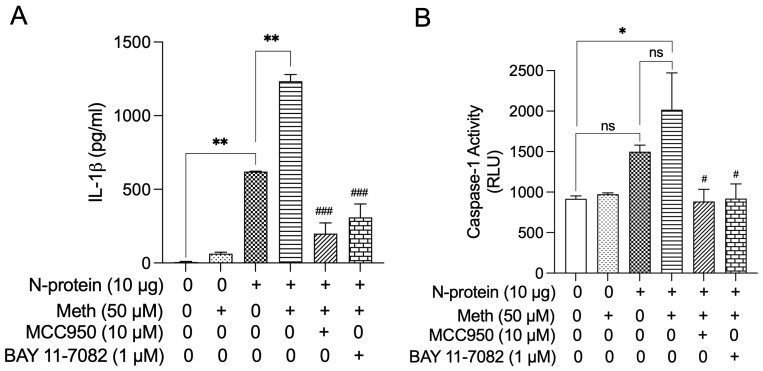
Meth promotes N-protein-induced cytokine production and caspase-1 activity. Concentration of secreted IL-1β (**A**) and the caspase-1 activity in the culture supernatants of microglial cultures (**B**). To confirm whether the secreted IL-1β and enhanced caspase-1 activity are mediated by the inflammasome via the NFκB pathway, we have included the NLRP3-specific inhibitor MCC950 (10 µM) and the NFκB pathway inhibitor BAY 11-7082 (1 µM). Microglial cultures were pretreated with the specified inhibitors for 2 h before N-protein treatment. ^ns^ *p* ≥ 0.05, * *p* < 0.05, and ** *p* < 0.01, vs. untreated control and N-protein-only treatment, and ^#^ *p* < 0.05, and ^###^ *p* < 0.001 vs. SARS-CoV-2 N-protein (10 µg/mL) and meth (50 µM) treatment.

IL-1β secretion in the culture supernatant induced by the SARS-CoV-2 N-protein was further significantly (*p* < 0.01) augmented by meth (50 µM) treatment (Figure 2A) [61,62]. Meth-induced augmentation of IL-1β secretion was significantly blocked by pretreatment of microglial cultures with the NLRP3-specific inhibitor MCC950 (*p* < 0.001) and the NFkB inhibitor BY11-7082 (*p* < 0.001) (Figure 2A), indicating the involvement of NLRP3 inflammasome activation via the NFkB pathway. Not only IL-1β secretion but also caspase-1 activity was found to be elevated upon meth treatment of N-protein-primed microglial cells (Figure 2B). Although the increase in caspase-1 activity in meth-treated cells compared to N-protein-primed cells was not significant compared to only N-protein-treated cells, there was a significant (*p* < 0.05) increase in caspase-1 activity when compared to the untreated control. Also, this meth-induced promotion of caspase-1 activity was blocked by pretreatment with MCC950 and BAY11-7082 (Figure 2B). Upon transcript analysis of the N-protein and meth treatments, we found higher expression of IL-1β, IL-6, TNF-α, and NLRP3 (Figure S2).

### 2.3. Meth Promotes N-Protein-Induced Inflammasome Processing

**Figure 3:**
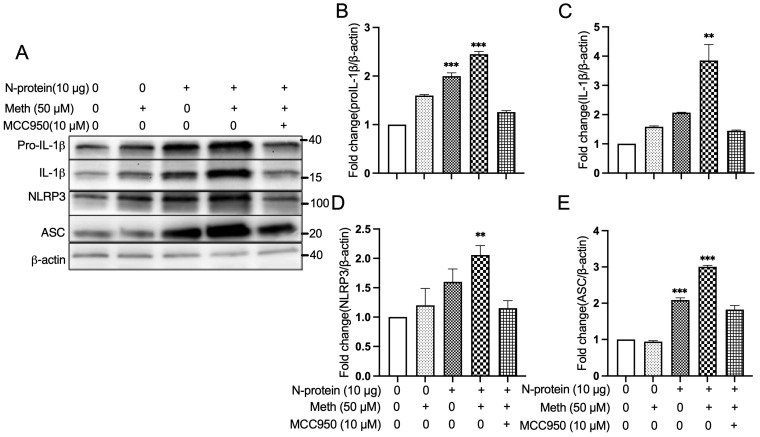
Effect of Meth on SARS-CoV-2 N-protein-induced expression of inflammasome components. The processing of IL-1β, NLRP3, and ASC in N-protein- and meth-treated rat microglial culture lysates was analyzed by Western blot (**A**). After standardization to β-actin, band densitometry was analyzed and quantified, as shown in the bar graphs. Pro-IL-1β (**B**), IL-1β (**C**), NLRP3 (**D**), and ASC (**E**) are in the respective bar graphs. ** *p* < 0.01, *** *p* < 0.001 vs. untreated control.

The expression of inflammasome components, including pro-IL-1β, NLRP3, and ASC, induced by SARS-CoV-2 N-protein, was measured at the protein level using Western blot analysis of microglial lysates (Figure S3). These expressions were normalized with β-actin and compared to the untreated control. We detected a significant increase in pro-IL-1β (*p* < 0.001), NLRP3 (*p* < 0.001), and ASC (*p* < 0.001) at 10 µg of N-protein treatment and a significant increase in IL-1β (*p* < 0.05) processing (Figure S3). Meth treatment further promoted the expression of pro-IL-1β, NLRP3, and ASC and the processing of IL-1β (Figure 3). Meth-promoted expression of pro-IL-1β, NLRP3, and ASC and the processing of IL-1β in N-protein-primed microglia were blocked by MCC950 (Figure 3), suggesting the involvement of the NLRP3 inflammasome.

### 2.4. NLRP3 and ASC Puncta in Meth and N-Protein-Induced NLRP3 Inflammasome

**Figure 4:**
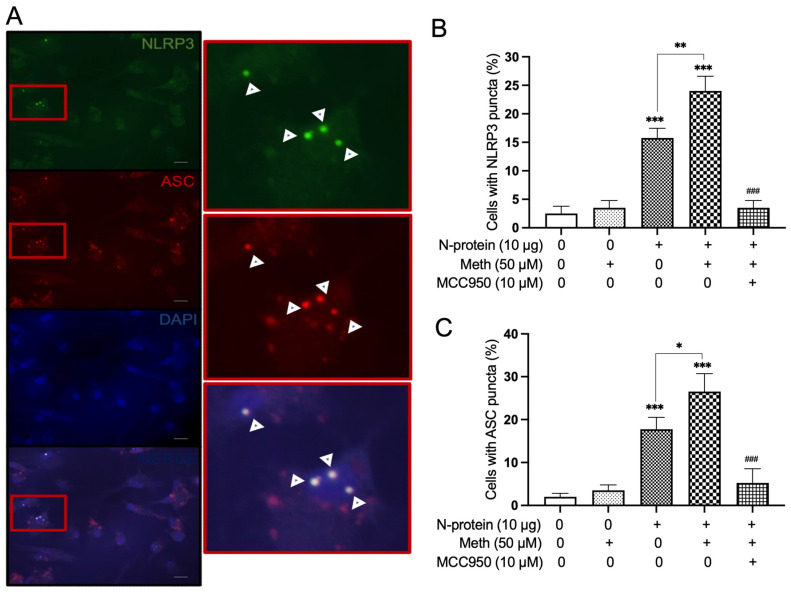
Meth enhances SARS-CoV-2 N-protein-induced NLRP3 inflammasome activation, as evidenced by NLRP3 and ASC puncta. Meth enhancement of SARS-CoV-2 N-protein-induced NLRP3 inflammasome activation was confirmed by quantifying NLRP3 and ASC puncta using immunofluorescence microscopy. PFA-fixed cells were stained with NLRP3 and ASC to monitor microglial NLRP3 activation (**A**). (**B**,**C**) Bar graphs showing the quantification results of NLRP3 and ASC puncta, respectively. Meth enhancement of SARS-CoV-2 N-protein-mediated inflammasome activation was significantly blocked by MCC950 (a specific NLRP3 inhibitor). Microglia were pretreated with 10 µM MCC950 2 h before N-protein treatment. *** *p* < 0.001 vs. untreated control, ** *p* < 0.01, and * *p* < 0.05 vs. only N-protein treatment, and ^###^ *p* < 0.001 vs. SARS-CoV-2 N-protein (10 µg/mL) and meth (50 µM) treatment. White triangles in microscopic images show puncta or psec. Scale bar = 30 µm. The appearance of NLRP3 and ASC puncta, and their colocalization, seems to overlap with nuclei; this is due to the three-dimensional nature of cells, where these events occur in the cytoplasm. While NLRP3 and ASC are not inside the nucleus, they are spatially associated with the nuclear envelope and often appear close to or overlapping with nuclear boundaries in microscopy images.

The hallmark of NLRP3 inflammasome activation is the appearance of NLRP3 and ASC puncta due to oligomerization and assembly of the inflammasome complex [30]. Furthermore, colocalization of NLRP3 and ASC puncta validates inflammasome activation. In the present study, using IFA to detect NLRP3 and ASC, we observed a significant increase in NLRP3 and ASC puncta, enhanced by N-protein treatment, as well as in NLRP3-ASC colocalization (Figure S4A). NLRP3 and ASC puncta were quantified by microscopic scoring of images [30], and a significant (*p* < 0.001) increase in NLRP3 and ASC puncta was found in SARS-CoV-2 N-protein treatments at both 1.0 and 10.0 µg (Figure S4B,C). MCC950 significantly (*p* < 0.001) blocked this N-protein-induced NLRP3 and ASC puncta formation and colocalization (Figure S4B,C). Interestingly, we found further enhancement of NLRP3 (*p* < 0.01) and ASC (*p* < 0.05) puncta formation, and of their colocalization, after meth treatment of N-protein-primed microglial cultures (Figure 4A–C). Furthermore, this enhancement of NLRP3 and ASC puncta formation, and their colocalization, was blocked by MCC950 (*p* < 0.001) (Figure 4B,C), suggesting a specific involvement of the NLRP3 inflammasome in meth augmentation of N-protein-primed microglial activation. Thus, it is evident that meth plays a critical role in promoting the microglial NLRP3 inflammasome in SARS-CoV-2-activated conditions. The appearance of NLRP3 and ASC puncta, and their colocalization, seems to overlap with nuclei. While NLRP3 and ASC are not inside the nucleus, they are spatially associated with the nuclear envelope and often appear close to or overlapping with nuclear boundaries in microscopy images.

### 2.5. NLRP3 and Caspase-1 Colocalization by Meth and N-Protein

**Figure 5:**
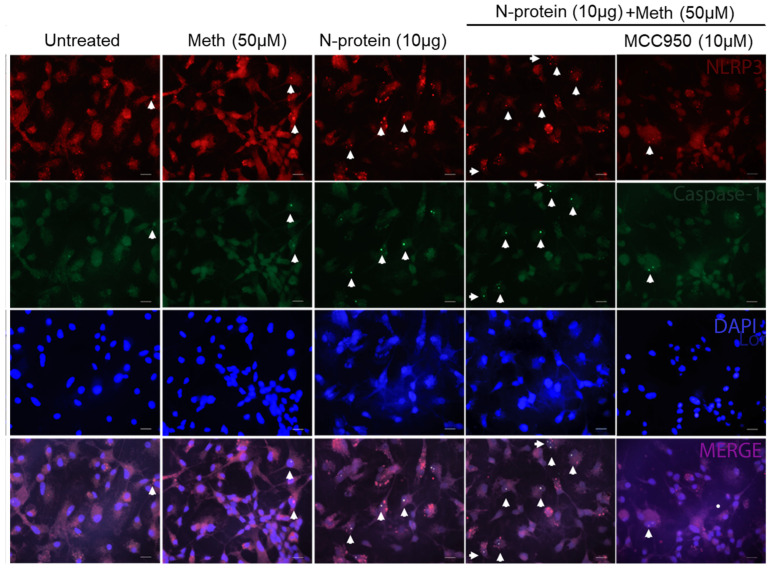
Meth further promotes NLRP3 and caspase-1 colocalization induced by SARS-CoV-2 N-protein. Meth enhancement of NLRP3 inflammasome activation was evident from enhanced NLRP3 and caspase-1 colocalization. The meth enhancement of SARS-CoV-2 N-protein-mediated inflammasome activation was blocked by MCC950 (a specific NLRP3 inhibitor). Microglial cultures were pretreated with MCC950 (10 µM) 2 h before N-protein treatment. The arrows in microscopic images show detection of specific proteins/enzymes involved. Scale bar = 30 µm.

To further confirm SARS-CoV-2 N-protein-mediated NLRP3 inflammasome activation, we examined the colocalization of NLRP3 and caspase-1 by IFA. We observed increased colocalization of NLRP3 and caspase-1 in microglial cultures treated with N-protein (Figure 5). This colocalization, which validates NLRP3 inflammasome activation, was further enhanced in meth-to-N-protein-primed microglial cultures (Figure 5). Pretreatment of microglial cells with MCC950 blocked colocalization of NLRP3 and caspase-1, further validating NLRP3 inflammasome activation and its promotion by N-protein and meth.

### 2.6. Meth Augmentation of N-Protein Enhances Outward K^+^ Current

**Figure 6:**
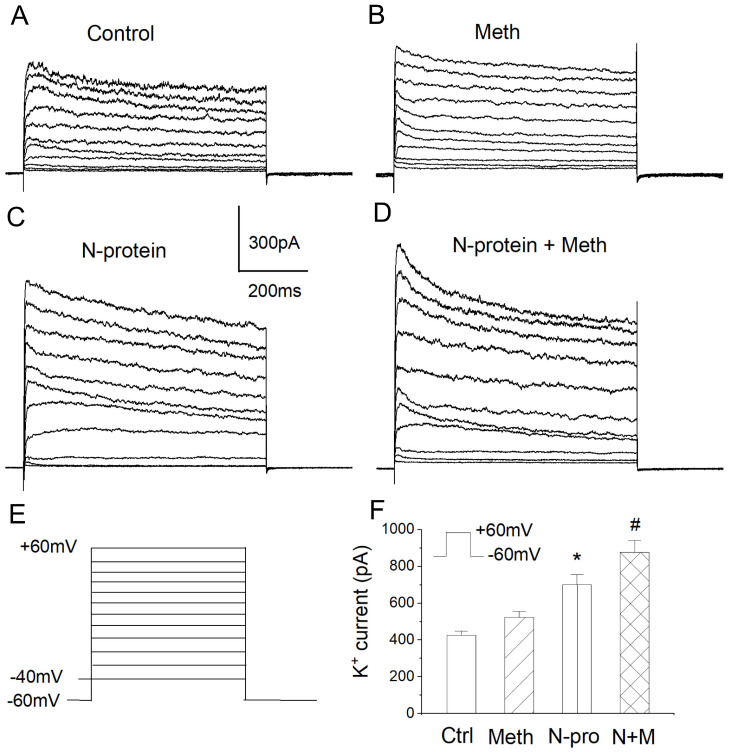
Meth augmentation of N-protein enhancement of outward K^+^ current. Representative current traces were recorded from microglial cells in the untreated control (**A**), pretreated with meth (**B**), pretreated with N protein (**C**), and pretreated with N protein + meth (**D**). (**F**) A summary bar graph illustrating the average peak outward K^+^ currents elicited by a voltage step from −60 to +60 mV in different experimental conditions. Note that meth per se had no significant effect on outward K^+^ current but augmented N protein enhancement of outward K^+^ current. The voltage protocol employed to generate outward current traces is shown in panel (**E**). * *p* < 0.05; ^#^ *p* < 0.05.

Electrophysiological studies of microglial cells have demonstrated that microglia express outward-rectifying K^+^ channels, specifically K_v_1.5 and K_v_1.3, the latter of which appears to be associated with microglial activation [63,64,65]. Exposure to a variety of activating stimuli produces a characteristic pattern of Kv1.3 upregulation [66,67]. We previously showed that meth potentiated gp120-induced neurotoxicity by augmenting gp120-induced increases in microglial outward K^+^ currents [68]. We hypothesize that meth might potentiate N-protein-associated neuroinflammation in microglial cells. To address this hypothesis, we tested the effects of meth and N-protein on microglial outward whole-cell K^+^ currents. Treatment of microglial cells with N-protein (10 µg) for 2 h produced an increase in outward K^+^ current, with an average peak current of 766.5 ± 56.1 pA (mean ± SD, *n* = 4) when the voltage step jumped from −60 mV to +60 mV (Figure 6). Compared with the average peak current of 425.3 ± 27.9 pA (*n* = 4) recorded from untreated microglial cells (control), the difference was statistically significant (*p* < 0.05). Treatment of microglia with meth (50 μM) had no significant effect on peak outward K^+^ current (482.1 ± 38.2 pA, *n* = 4) when compared with the peak outward K^+^ current recorded from microglia in controls (Figure 6). However, the peak outward K^+^ current recorded from microglia treated with meth and N-protein in combination was 877.5 ± 63.7 pA (*n* = 4), a significant increase when compared with the peak outward K^+^ current recorded from microglia treated with N-protein alone (*p* < 0.01), demonstrating an augmentative effect of meth on the N-protein-induced increase in outward K^+^ currents in microglial cells (Figure 6). Due to increased K^+^ efflux induced by SAES-CoV-2 N-protein and further enhancement by meth, intracellular K+ levels are lower^,^ which permits NLRP3 conformational changes and facilitates NLRP3 inflammasome complex formation, leading to NLRP3 inflammasome activation. Hence, SARS-CoV-2 N-protein combined with meth enhances microglial NLRP3 inflammasome activation.

### 2.7. Sigma-1 Receptor (σ1-R) in Meth-Mediated N-Protein-Primed Microglial NLRP3 Inflammasome Activation

**Figure 7:**
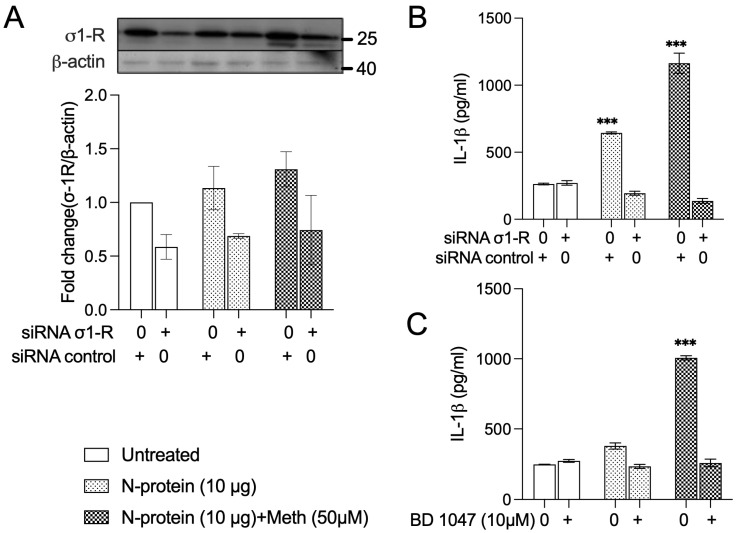
Sigma-1 receptor in meth enhancement of SARS-CoV-2 N-protein-induced microglial NLRP3 inflammasome activation. The role of the sigma-1 receptor (σ1-R) in N-protein and meth enhancement of NLRP3 inflammasome activation was assessed by measuring σ1-R expression and IL-β secretion after σ1-R-specific siRNA transfection and σ-1R-specific inhibitor (BD 1047) pretreatment. Microglial cultures were pretreated with BD 1047 (10 μM) 2 h before N-protein treatment or transfected with anti-sigma-1 specific siRNA for 48 h before N-protein treatment. Cell lysates were subjected to Western blot analysis, and the culture supernatant was analyzed by ELISA to measure the released IL-β. (**A**) σ1-R expression after siRNA transfection, (**B**) concentration of IL-1β in culture supernatant after siRNA transfection, and (**C**) concentration of IL-1β in culture supernatant after BD 1047 pretreatment. *** *p* < 0.001 vs. untreated control.

Although the expression of NLRP3 and IL-1β was enhanced after meth treatment of N-protein-treated microglial cultures, there was also increased expression of TLR4 and σ1-R. However, downstream components, such as NF-κB and IκBα, increased in response to N-protein treatment. Intriguingly, their expression decreased upon meth treatment in N-protein-primed microglia, suggesting the involvement of pathways beyond the traditional TLR4-NF-κB-IκBα pathways in meth’s promotion of NLRP3 inflammasome activation. To explore the mechanism by which meth augmentation of SARS-CoV-2 N-protein-induced microglial activation is mediated via the NLRP3 inflammasome, we investigated the involvement of the opioid receptor sigma-1 (σ-1R). We observed increased σ-1R expression in microglial cell lysates treated with the SARS-CoV-2 N-protein, as determined by immunoblotting. Meth treatment of these N-protein-treated microglial cells further enhanced σ1-R expression (Figure S5). To confirm meth exacerbation of N-protein-induced microglial NLRP3 activation, we blocked σ-1R. To block the effect of σ1-R, we employed two parallel approaches: one involved genetic knockdown of σ1-R, and the second utilized the σ1-R-specific inhibitor BD 1047. To knock down σ-1R, we used an siRNA approach and transfected microglial cultures for 48 h before treatment. We observed a significant reduction in σ-1R in siRNA-transfected microglial cultures following N-protein and combined N-protein and meth treatment (Figure 7A). There were no increases in IL-1β secretion observed in either N-protein- or combined N-protein and meth-treated microglial cultures transfected with anti-σ1-R siRNA (Figure 7B). However, there was a significant increase in IL-1β secretion in microglial cultures transfected with scrambled siRNA and treated with either N-protein or with a combination of N-protein and meth. Thus, it was evident that σ-1R plays a pivotal role in the meth promotion of N-protein-induced microglial NLRP3 inflammasome activation, resulting in microglial activation.

Further, to confirm the critical role of σ-1R in meth augmentation of N-protein-induced microglial NLRP3 inflammasome activation, we used the σ-1R-specific inhibitor BD 1047. Pretreatment of microglial culture with BD 1047 two hours before N-protein or N-protein and meth treatment resulted in no increase in IL-1β secretion in culture supernatants (Figure 7C). Thus, treatment with the σ-1R-specific inhibitor BD 1047 reconfirms our observation of the crucial role of σ-1R in meth exacerbation of N-protein-induced microglial NLRP3 inflammasome activation.

### 2.8. Meth Augmentation of N-Protein-Induced Microglial iNOS Expression and NO Production

**Figure 8:**
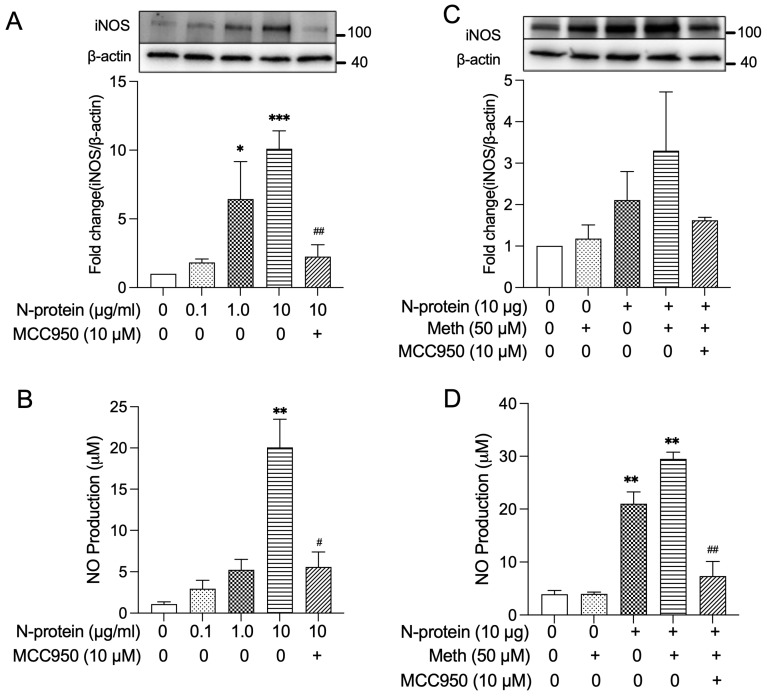
Meth promotes SARS-CoV-2 N-protein-induced microglial iNOS expression and NO production. The SARS-CoV-2 N-protein induces inducible nitric oxide synthase (iNOS) expression and nitric oxide (NO) production in microglia. N-protein-induced iNOS expression and NO production were enhanced by meth. Rat microglial cultures treated with N-protein and meth were subjected to Western blot analysis. After normalization to β-actin, band densitometry was analyzed and quantified, as shown in bar graphs (**A**,**C**). The collected culture supernatant showed enhanced NO levels after N-protein treatment (**B**), which were further enhanced upon meth treatment (**D**). The SARS-CoV-2 N-protein induced iNOS expression and NO production, and the meth exacerbation was blocked by MCC950 (NLRP3-specific inhibitor). * *p* < 0.05, ** *p* < 0.01, *** *p* < 0.001 vs. untreated control. ^#^ *p* < 0.05 ^##^ *p* < 0.01, vs. SARS-CoV-2 N-protein (10 µg/mL) treatment in (**A**, **B**) and vs. SARS-CoV-2 N-protein (10 µg/mL) and meth (50 µM) treatment in (**C**, **D**).

We observed that treating primary rat microglial cultures with N-protein increased iNOS expression in a dose-dependent manner (Figure 8A). Pretreating microglial cultures with MCC950 blocked iNOS expression. Additionally, culture supernatants from N-protein-treated microglia showed increased nitric oxide (NO) production (Figure 8B). Both iNOS expression and NO production were further augmented by meth in N-protein-treated microglial cultures (Figure 8C,D). The iNOS-mediated release of neurotoxic NO may influence neuroinflammation and neurodegeneration. Thus, there is potential for NO-mediated exacerbation of long COVID in people abusing drugs, such as meth.

## 3. Discussion

SARS-CoV-2 is the causative agent of COVID-19, and all efforts were focused on controlling its spread and the pandemic. The discovery of a vaccine, its emergency use authorization, and the repurposing of antiretrovirals have finally brought this pandemic to an end, nearly three years after the onset of COVID-19. SARS-CoV-2 is primarily a respiratory pathogen, but it also causes multiorgan infections, including those affecting the CNS [69,70,71,72]. Although the worst phase of SARS-CoV-2/COVID-19 is over, debilitating long-term post-COVID-19 effects from COVID-19-mediated neurological manifestations, known as long COVID or PASC, remain a global concern [26,72,73,74,75,76,77,78]. These neurological manifestations in COVID-19 result from direct viral invasion of the CNS via disruption of the BBB or retrograde transport along olfactory neurons [21,22,23,79]. Furthermore, SARS-CoV-2 or neurotoxic viral proteins in the CNS may induce neurological manifestations. Among these viral proteins, reports indicate that S, N, E, and viroporins have such neurotoxic consequences [57,80,81,82]. In the present study, we investigated SARS-CoV-2 N-protein-mediated neurological manifestations and the impact of methamphetamine abuse. N-protein is a small structural protein and an abundant antigen used for disease testing; it is resistant to mutations, unlike the S-protein. We chose rat microglia as our cellular model to investigate neuroinflammation and subsequent neurodegeneration, which may lead to neurocognitive outcomes that contribute to the development of long COVID or PASC. Rat microglia serve as a simpler cellular model to study SARS-CoV-2-mediated neuroinflammation and neurodegeneration. Though there is no evidence that rat microglia express the ACE2 receptor protein for SARS-CoV-2 infection, there are studies showing the effects of various SARS-CoV-2 proteins on microglial NLRP3 inflammasome activation. Several studies have demonstrated SARS-CoV-2 neuroinvasion and COVID-19-mediated neuroinflammation, as well as the resulting neurodegeneration [57,59,60,79,83]. Additionally, because SARS-CoV-2 does not directly infect neurons due to the lack of or low-level expression of the ACE2 receptor, viral protein-mediated neuroinflammation may occur indirectly via microglial activation. Microglia are typically activated in response to injury to protect against the insult. Microglia possess specialized innate immune sensors, including NLRP3, which detect pathogen-associated molecular patterns (PAMPs) and damage-associated molecular patterns (DAMPs). The resulting insults trigger excessive cytokine secretion via NLRP3 inflammasome activation, which in turn leads to neurotoxicity, including neuroinflammation and neurodegeneration [38,84,85,86].

Drug abuse further promotes this SARS-CoV-2-mediated neuroinflammation and neurodegeneration by NLRP3 inflammasome activation [55], but the exact mechanism is unknown. In the present study, we investigated the molecular mechanisms underlying meth augmentation of SARS-CoV-2-mediated inflammasome activation, which leads to neuroinflammation and neurodegeneration. The study was initiated with N-protein treatment in primary microglial cultures from neonatal rats, followed by the measurement of proinflammatory cytokine release in the culture supernatant. These microglial cultures responded to N-protein treatment with dose-dependent cytokine release (Figure S1). As the cytokine release was successfully blocked by the NLRP3-specific inhibitor MCC950, we speculated that this N-protein-mediated cytokine release was due to NLRP3 inflammasome activation. Additionally, the caspase-1 activity assay yielded encouraging results and supports our indication of NLRP3 inflammasome involvement in this (Figure S1). We included LPS (100 and 500 ng/mL) in these experiments as a positive control. To explore the involvement of the NLRP3 inflammasome, we measured the processing of its components and products. The densitometric analysis of the Western blots of microglial lysates treated with N-protein revealed a dose–response in the protein expression of pro-IL-1β, IL-1β, NLRP3, and ASC (Figure S3). What was odd in this N-protein-mediated inflammasome activation was the expression of ASC. As earlier studies of NLRP3 inflammasome activation have not shown ASC expression changes with treatment, N-protein may be acting in a distinct way and warrants dedicated investigation. Similarly, the addition of meth to N-protein-primed microglial cultures led to increased IL-1β secretion and caspase-1 activity (Figure 2). These meth-mediated increases in IL-1β secretion and caspase-1 activity were further blocked by MCC950 and BAY11-7082, suggesting involvement of the NLRP3 inflammasome and activation via the NF-κB pathway. The processing of components and products was further analyzed to provide more detail on inflammasome activation. There was a significant increase in the expression of proIL-1β, IL-1β, NLRP3, and ASC (Figure 3). Further, Iba1 staining of microglial cultures showed that N-protein induced microglial activation, and this N-protein-induced microglial activation was augmented by meth treatment (Figure 1). Pretreatment of MCC950 blocked microglial activation; this suggests that N-protein-mediated microglial activation is via NLRP3 inflammasomes. NLRP3 inflammasome activation occurs through the oligomerization of NLRP3, ASC, and caspase-1; this oligomerization forms structures called specks or puncta that can be visualized after staining with antibodies specific to NLRP3 and ASC. These specks, or puncta, are considered hallmarks of NLRP3 inflammasome activation, and colocalization of NLRP3 and ASC specks further validates inflammasome activation. In the present study, we found increased NLRP3 and ASC speck or puncta formation upon N-protein treatment of microglial cultures (Figure S4). We further observed significant dose-dependent increases in NLRP3 and ASC puncta, which overlapped and colocalized. Upon microscopic scoring, we observed increments in ASC and NLRP3 puncta following meth treatment of N-protein-primed microglial cultures, with increased overlap and colocalization of these puncta, suggesting meth augmentation of NLRP3 inflammasome activation (Figure 4). As pretreatment with MCC950 blocked the formation of microglial NLRP3 and ASC puncta, meth’s promotion of N-protein-mediated microglial activation may occur via NLRP3 inflammasomes. Furthermore, N-protein treatment of microglial cultures increased colocalization of NLRP3 and caspase-1, and meth further augmented this colocalization (Figure 5).

To investigate the mechanism, we employed an electrophysiological approach and observed increased K^+^ efflux in microglial cultures following N-protein treatment (Figure 6). This K^+^ efflux was further enhanced by meth treatment, suggesting that meth promotes NLRP3 activation, at least in part, through potassium channels. Thus, the K^+^ channel appears to be involved in the NLRP3 inflammasome activation mechanism in response to N-protein treatment and meth exacerbation. To investigate the role of other factors in this mechanism of meth augmentation of NLRP3 inflammasome activation, we examined the TLR4 receptor. We found increased TLR4 upon N-protein treatment, but no further enhancement upon meth treatment. This was followed by increased NFκβ and Iκκβa expression with N-protein, but decreased expression upon meth treatment in these N-protein-primed cells. Furthermore, TLR2 may be involved in NLRP3 inflammasome activation. So, together, TLR2 and TLR4 may provide the priming signal (signal-1) for NLRP3 inflammasome activation in the presence of SARS-CoV-2 N-protein and meth. Also, these signals may activate the NFκβ pathway, leading to N-protein-induced inflammasome activation using activation signal (signal −2) via K^+^ efflux. Given the lack of evidence for a surface receptor for N-protein binding in rat microglial cells, this may occur via immune activation via other receptors, K^+^ efflux enhancement, or reactive oxygen species (ROS). Furthermore, the meth augmentation may affect the NLRP3 inflammasome via opioid receptors. We investigated the sigma-1 receptor (σ1-R) in the meth augmentation of the NLRP3 inflammasome. The expression of σ1-R increased upon N-protein treatment and was further increased upon meth treatment of N-protein-primed cells. This σ1-R expression was significantly blocked (*p* < 0.001) by MCC950 and by the σ1-R-specific inhibitor BD1047 (*p* < 0.001). This experiment suggests that σ1-R plays a role in the meth-promoted N-protein-induced NLRP3 inflammasome activation. Furthermore, to validate the involvement of σ1-R in NLRP3 inflammasome activation, we employed two parallel approaches: siRNA against σ1-R and the chemical inhibitor BD1047 to nullify its effect. Overall, siRNA treatment decreased σ1-R expression compared to the scrambled negative control siRNA (Figure 7). Cytokine secretion by microglial cells treated with scrambled negative control siRNA increased upon N-protein treatment and further increased upon meth treatment in these N-protein-treated cells. However, when microglial cells were treated with siRNA against σ1-R, no increase in IL-1β secretion was observed upon N-protein treatment or meth treatment of N-protein-primed cells. This suggests that conditional knockdown of σ1-R results in the ablation of cytokine secretion and thus that meth promotes NLRP3 inflammasome activation via σ1-R signaling. Similar results were observed when microglia were pretreated with BD1047, a specific σ1-R inhibitor (Figure 7C). Thus, these results suggest the involvement of σ1-R in meth augmentation of N-protein-treated microglial NLRP3 inflammasome activation, causing neuroinflammation. Further research may validate the crucial role of meth in promoting NLRP3 inflammasome activation and may provide a solution to control drug addiction-mediated neuroinflammation. As σ1-R is abundant in mitochondria and the endoplasmic reticulum (ER), these organelles may have a fine-tuned, mechanistic involvement in this meth promotion. Future investigations using model organisms may reveal intricate mechanisms.

**Figure 9:**
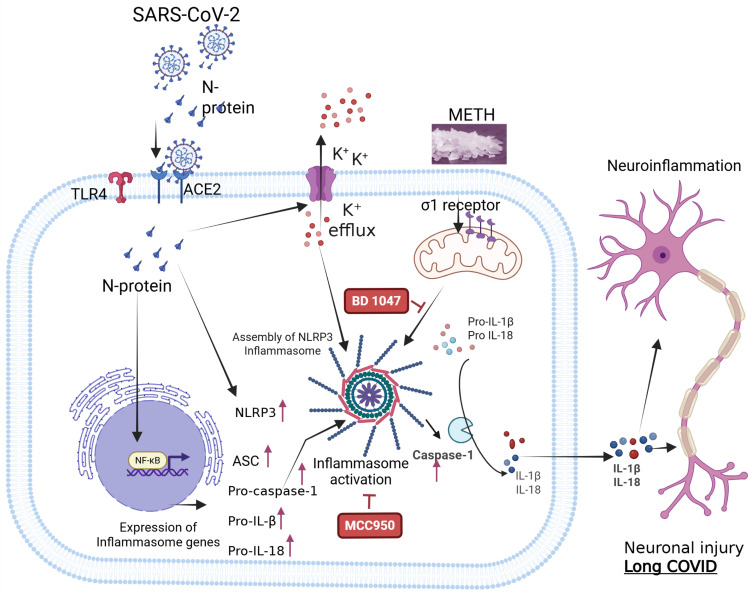
Schematic diagram illustrating the mechanisms involved in NLRP3 inflammasome in meth- and SARS-CoV-2 N-protein-mediated neuroinflammation. SARS-CoV-2 N-protein released during SARS-CoV-2 infection causes NLRP3 inflammasome activation via NFkB signaling. Meth enhances NLRP3 inflammasome activation by the SARS-CoV-2 N-protein via the sigma-1 receptor. The SARS-CoV-2 N-protein and meth cause neuroinflammation by increasing proinflammatory cytokine release through NLRP3 inflammasome activation, leading to neurodegeneration and exacerbating long COVID. Black arrows show the action, and red arrows show increased levels of proteins, cytokines, and enzymes, and the red T sign shows inhibitory action by either BD1047 or MCC950. This figure was created in BioRender. Debashis Dutta (2026) https://app.biorender.com/illustrations/66bcf8591ffa4f59e84bf83f (accessed on 6 May 2026).

Neurotoxicity, characterized by neuroinflammation and neurological manifestations, was observed in N-protein-treated microglia, with meth further augmenting N-protein-primed microglia, as measured by iNOS expression and NO production (Figure 8). We observed increased iNOS expression and NO production in microglial cultures after N-protein treatment, and these responses were further enhanced by meth treatment in N-protein-treated cells. Thus, these results suggest that N-protein can enhance neurotoxic NO production, along with proinflammatory cytokines and chemokines, and that meth abuse further amplifies these effects. Overall, the present study revealed that drug abuse, particularly of meth, could augment neurological manifestations in COVID-19 patients (Figure 9). These effects can exacerbate the impact of the imminent silent killer, long COVID or PASC. Thus, consideration of inflammasome inhibitors is warranted to address the complex issue of long COVID. However, a limitation in interpreting these results is the study’s in vitro design, and further in vivo validation may be required in future studies.

## 4. Materials and Methods

### 4.1. Materials

Recombinant human coronavirus SARS-CoV-2 nucleocapsid (N-protein) was obtained from Thermo Fisher (Cat # RP-87665, Invitrogen, Rockford, IL, USA). The protein was resuspended in sterile water for functional studies. Methamphetamine was purchased from Sigma-Aldrich (St. Louis, MO, USA; Cat # M-8750) under DEA license # RX0374974. Lipopolysaccharide (LPS; from *Escherichia coli* 0111:B4; Cat # L4391) was also obtained from Sigma-Aldrich (St. Louis, MO, USA). MCC950 (Cat # 538120) was obtained from Enzo Life Sciences (Farmingdale, NY, USA). All other chemicals, unless otherwise specified, were purchased from Sigma-Aldrich.

### 4.2. Animals

Pregnant female Sprague-Dawley (SD) rats were purchased from Charles River Laboratories (Wilmington, MA, USA) for primary microglia isolation from neonates. These pregnant animals were housed in the university animal facility at a constant temperature (22 °C) and relative humidity (50%), under a regular light–dark cycle (light on at 7:00 a.m. and off at 5:00 p.m.), with round-the-clock access to adequate food and water. All animals used in the study and the procedures were strictly reviewed by the Institutional Animal Care and Use Committee (IACUC) of the University of Nebraska Medical Center (IACUC No. 19-085-07-FC).

### 4.3. Methods

#### 4.3.1. Isolation and Culture of Primary Microglia from Neonatal Rats

Microglial cells were isolated from the cerebral cortex of postnatal (0–1-day-old) SD rats using previously described procedures [87]. Briefly, rat cortical tissues were dissected in cold Hank’s Balanced Salt Solution (HBSS; Cat # 14025-076; Life Technologies, Grand Island, NY, USA) and digested with 0.25% trypsin (Cat # 25200-056) and 200 Kunitz units/mL DNase (Cat # D4263; Sigma-Aldrich, St. Louis, MO, USA) at 37 °C for 30 min. The digested tissues were suspended in cold HBSS and filtered through 100 µm and 40 µm pore-size cellular strainers (Cat # 542000 and 542040; Greiner Bio-One GmbH, Frickenhausen, Germany), respectively. For microglial culture, the isolated cells (25 × 10^6^) were plated into T75 cm^2^ flasks (Cat # 90076; TPP, Techno Plastic Products AG, Trasadingen, Switzerland) in high-glucose Dulbecco’s modified Eagle’s medium (DMEM; Cat # 11965-084; Life Technologies, Grand Island, NY, USA) containing 10% fetal bovine serum (FBS; Cat # A5670701; Thermo Fisher Scientific, Asheville, NC, USA), 1×glutaMAX (Cat # 35050; Life Technologies, Grand Island, NY, USA), 1% penicillin/streptomycin (Cat # 30-002-Cl; Mediatech, Inc., Manassas, VA, USA), and 300 ng/mL macrophage colony-stimulating factor (M-CSF) supplied by the Department of Pharmacology and Experimental Neuroscience, University of Nebraska Medical Center. After 8–10 days in culture, the flasks were gently shaken, and detached cells were collected and seeded onto 6-well (2 × 10^6^/well), 12-well (1 × 10^6^/well), or 96-well (0.5 × 10^6^/well) plates, based on experimental requirements, using M-CSF-free DMEM complete media. Suspended glial cells were removed 1 h after seeding by replacing the culture medium. The resulting cultures contained >98% microglia, as determined by staining with anti-CD11b (Cat # ab8879; Abcam, Cambridge, MA, USA), a microglial marker.

#### 4.3.2. Determination of Cytokine Production by Enzyme-Linked Immunosorbent Assay (ELISA)

Secretion of IL-1β and other cytokines in the culture supernatant was measured by ELISA. Microglia were primed with N-protein for 48 h and then stimulated with the indicated concentration of meth. After 48 h of priming with N-protein, the microglia were washed before meth treatment. For the detection of IL-1β release, the supernatant of meth-treated microglia was collected at 24 h post-meth treatment. Cytokines in supernatants were detected using the ELISA kit (Cat # DY501 for IL-1β, DY510 for TNFα, DY521-05 for IL-18, and DY506 for IL-6; R&D Systems, Inc., Minneapolis, MN, USA). The experiments were performed according to the manufacturer’s instructions. Briefly, the plates were coated overnight at room temperature with a capture antibody diluted in reagent dilution buffer. After overnight incubation, the plates were washed three times with 1× wash buffer. The plates were then blocked with a diluted reagent buffer to prevent nonspecific antigen binding. The capture antibody-coated and blocked 96-well plates were incubated with collected supernatants for 2 h at room temperature, followed by a 2 h incubation with the detection antibody. Finally, the Streptavidin-HRP working solution was incubated for 20 min, after which the substrate solution was added to each well. After the reaction was stopped with a stop solution and the reading was performed with a microplate reader (Bio-Rad, Hercules, CA, USA) using filters at 450/560 nm, the result was calculated using a 4-parameter curve fit.

#### 4.3.3. Immunoblots

After priming with N-proteins for 48 h, microglial cells were treated with meth for an additional 24 h. After treatment with a combination of N-protein and meth, cells were lysed in RIPA buffer (Cat # 89901; Thermo Fisher Scientific, Asheville, NC, USA) for analysis of pro-IL-1β processing. In most cases, approximately 30 µg of total protein was separated on a 4–20% gradient PAGE gel (Cat # 4561094; Bio-Rad, Hercules, CA, USA) and transferred to polyvinylidene difluoride (PVDF) membranes (Cat # 1620177; Bio-Rad, Hercules, CA, USA). Membranes were blocked with 3% bovine serum albumin (BSA; Cat # A3059; Sigma-Aldrich, Saint Louis, MO, USA) in 1X tris-buffered saline (TBS) and incubated overnight at 4 °C with rabbit polyclonal antibody for IL-1β (Cat # EPR21086; Abcam, Cambridge, MA, USA) at 1:500 dilution, mouse polyclonal antibody for NLRP3 at 1:1000 dilution (Cat # AG-20B0014-C100; AdipoGen, San Diego, CA, USA), rabbit Iba-1 antibody (Cat # 016-20001; Fujifilm, Richmond, VA, USA) at 1:1000 dilution, or anti-mouse β-actin monoclonal antibody (1:5000; Cat # A5441; Sigma-Aldrich, Saint Louis, MO, USA). The washing buffer was 1X TBS with 0.2% Tween (TBS-T). For iNOS expression, a rabbit polyclonal antibody for iNOS (Cat # ab15323; Abcam, Cambridge, MA, USA) was used at a 1:500 dilution. The secondary antibody was horseradish peroxidase (HRP)-conjugated anti-rabbit (Cat # 31460; Invitrogen, Rockford, IL, USA) or anti-mouse (Cat # 31430; Invitrogen, Rockford, IL, USA). Labeled proteins were visualized using the Pierce-enhanced chemiluminescence (ECL) system (Cat # 30496; Thermo Fisher Scientific, Waltham, MA, USA).

#### 4.3.4. Immunofluorescence Microscopy

Immunocytochemistry was performed to assess microglial activation and quantify NLRP3 puncta as a readout of inflammasome activation. Microglia were seeded onto coverslips in a 24-well plate at 0.5 × 10^6^ cells per well. After priming with N-protein (10 μg) for 48 h, meth treatment was administered for 24 h. Iba-1 staining was performed to monitor microglial activation. At room temperature, cells were fixed with 4% paraformaldehyde (PFA) (Cat # J19943-K2, Janssen Pharmaceuticals 3a, Geel, Belgium) for 10 min. Cells were then blocked and permeabilized in PBS containing 10% normal goat serum (Cat # S-1000; Vector Laboratories, Inc., Newark, CA, USA) and 0.1% Triton X-100 for 15 min. Primary antibodies included a mouse polyclonal NLRP3 antibody (Cat # sc-518122; Santa Cruz, CA, USA) and a rabbit Iba-1 antibody (Cat # sc-32725; Santa Cruz, Dallas, TX, USA) at 1:100 dilution. Rabbit polyclonal antibodies for ASC (Cat # sc-22514-R; Santa Cruz, Dallas, TX, USA) at 1:200 and caspase-1 (Cat # sc-514; Santa Cruz, Dallas, TX, USA) at 1:500 were used as needed. Microglia were identified using a mouse monoclonal CD11b antibody at 1:500 (Cat # ab8879; Abcam, Cambridge, MA, USA). Secondary antibodies were goat anti-rabbit Alexa 488 (1:1000, Cat # A-11034) and Alexa594 (1:1000, Cat # A-11012), and goat anti-mouse Alexa 488 (1:1000, Cat # A-11001) and Alexa594 (1:1000, Cat # A-11032) from Thermo Fisher Scientific (Waltham, MA, USA). All fluorescence images were acquired under identical imaging conditions. Representative images shown in the figures were adjusted uniformly for visualization purposes only.

#### 4.3.5. qRT-PCR Measurement of Cytokine Transcript

Following the manufacturer’s protocol, total RNA from microglial cultures was isolated using the RNeasy Mini Kit (Cat # 74104; Qiagen, Germantown, MD, USA). RNA concentration and purity were measured using a NanoDrop 2000 spectrophotometer (Thermo Fisher Scientific). In total, 1 μg of total RNA was used for cDNA synthesis with the cDNA Reverse Transcriptase Kit (ThermoFisher). Quantitative Real-Time PCR (qRT-PCR) was performed on an Applied Biosystems Real-Time PCR System using SYBR Green Master Mix (both from Thermo Fisher). Data were analyzed using the 2^−ΔCt^ method, with glyceraldehyde 3-phosphate dehydrogenase (*GAPDH*) as the control. A list of primers used in this qRT-PCR is provided in Table S1.

#### 4.3.6. SiRNA Transfection

We procured siRNA for the sigma-1 receptor (Catalog #AM16708) and its negative control, scrambled siRNA (Catalog #AM4611), from Thermo Fisher Scientific. siRNA transfection was performed using Lipofectamine™ RNAiMAX transfection reagent (Cat # 13778075, Thermo Fisher Scientific) 48 h prior to SARS-CoV-2 N-protein treatment. Briefly, 5 nmol of siRNA was transfected into each well containing 1 × 10^6^ microglia in serum-free media. The transfection medium was replaced after 24 h with fresh DMEM supplemented with 10% FBS, and the cells were incubated for an additional 24 h. Later, the cells were treated with SARS-CoV-2 N-protein for 48 h. After 48 h of incubation with N-protein, cells were washed with PBS and treated with methamphetamine for another 24 h. After final incubation, culture supernatant was collected for ELISA, and cellular lysate was collected for Western blot.

#### 4.3.7. Caspase-1 Inflammasome Assay

We used the Caspase-Glo 1 inflammasome assay kit (Cat # G9951; Promega, Madison, WI, USA) to measure caspase-1 activity in the culture supernatant. The assay was performed according to the manufacturer’s protocol. Briefly, 50 µL of culture supernatant was transferred to a 96-well plate, and 50 µL of Caspase-Glo 1 was added to each well. The mixture was gently shaken on a plate shaker at 300–500 rpm for 30 s. The plate was incubated at room temperature for 60 min to stabilize the luminescent signal. Afterward, luminescence was measured using a microplate reader.

#### 4.3.8. Electrophysiology

Outward K^+^ currents were recorded from primary rat microglial cultures at room temperature using whole-cell voltage-clamp techniques. Pre-treated microglia were perfused with oxygenated artificial cerebrospinal fluid (ACSF) bubbled with 95% O_2_, 5% CO_2_, containing (in mM) NaCl 150, KCl 4.5, CaCl_2_ 2, MgCl_2_ 1, HEPES 5, glucose 11, pH 7.3 (adjusted with NaOH), with an osmolarity of 310 mOsm. Patch-clamp electrodes, pulled from borosilicate glass capillaries (WPI, Sarasota, FL, USA), had a tip resistance of 4–6 MΩ when filled with pipette solution containing the following (mM): KCl 150, MgCl_2_, CaCl_2_ 1, EGTA 11, HEPES 10, with a pH of 7.3 adjusted with KOH. Whole-cell currents were evoked by voltage steps (600 ms in duration), starting from a holding potential of −60 mV, then to −40 mV, and finally to +60 mV in 10 mV increments. The seal resistance was >1 GΩ. Junction potentials were corrected, and the cell capacitance was compensated (~70%). Current signals were amplified with an Axopatch 200B amplifier (Molecular Devices, Sunnyvale, CA, USA). The current and voltage traces were displayed and recorded on a Dell computer using the pClamp 10 data acquisition and analysis system. Peak outward K^+^ currents were measured and analyzed.

#### 4.3.9. Measurement of NO Production

Nitrite concentrations in the culture supernatant were measured using the Griess reagent system (Cat # TB229; Promega, Madison, WI, USA) according to the manufacturer’s instructions. After treatment with N-protein and meth, 50 µL aliquots of culture supernatant were collected from each treatment group. Then, 50 µL of sulfanilamide solution and 50 µL of NED solution were added to the collected supernatants, and the mixtures were incubated for 10 min. The absorbance of the final samples was measured using a microplate reader (Bio-Rad, Hercules, CA, USA) with a 560 nm filter.

#### 4.3.10. Data Analyses

All data are expressed as means ± SDs unless otherwise indicated. Statistical analyses were performed using one-way ANOVA with Tukey’s multiple-comparison post hoc test (GraphPad Prism, version 11.0.2). A *p*-value of 0.05 or less was used as the significance level for all tests. The densities of the target Western blot bands were quantified using NIH ImageJ (Java 1.8.0_172, 64-bit) and normalized to the β-actin band density. The percentage of microglia with NLRP3 puncta was estimated by microscopic scoring, as described in the previous article by Rodrigues et al. All experiments were performed in technical and biological triplicate unless otherwise specified.

## 5. Conclusions

Our present study showed that SARS-CoV-2 N-protein-mediated activation of the NLRP3 inflammasome in rat microglial cultures may contribute to CNS cytokine storm and COVID-19-mediated neurological complications. In addition to previously reported NLRP3 inflammasome activation by SARS-CoV-2 infection and viral proteins, we demonstrated that the microglial NLRP3 inflammasome can be activated by the SARS-CoV-2 N-protein. In this study, we present evidence of N-protein-mediated activation of the microglial NLRP3 inflammasome in primary rat microglial cultures. This study further indicates that methamphetamine augments SARS-CoV-2 N-protein-mediated NLRP3 inflammasome activation in microglial cultures, thereby amplifying neuroinflammation, leading to neurological consequences, and further exacerbating long COVID or PASC. Thus, methamphetamine-abuse-mediated NLRP3 inflammasome exacerbation may further amplify neuroinflammation and impose a higher toll on long COVID. Furthermore, using the NLRP3 inflammasome inhibitor MCC950, the sigma-1 receptor inhibitor BD 1047, and siRNA against the sigma-1 receptor, we have demonstrated successful control of inflammatory mediators. Hence, targeting NLRP3 inflammasome-mediated inflammation in SARS-CoV-2-infected individuals will help combat neuronal damage and neuroinflammation, and these inhibitors could serve as potential therapeutics for long COVID. Thus, the results provide plausible targets for therapeutic intervention for lingering long COVID, which is applicable to drug-abusing individuals as well. Future in vivo studies may further validate our in vitro results and open an avenue for a therapeutic strategy to intervene in long COVID.

## Data Availability

The original contributions presented in this study are included in the article/Supplementary Materials. Further inquiries can be directed to the corresponding authors.

## Supplementary Materials

The following supporting information can be downloaded at: https://www.mdpi.com/article/10.3390/ijms27156960/s1.
