# Supplementary material for: Involvement of NLRP3 Inflammasome in Methamphetamine Augmentation of SARS-CoV-2 N-Protein-Induced Neuroinflammation in Rat Microglial Cells
Source: Int J Mol Sci. 2026 Aug 3;27(15):6960. doi: 10.3390/ijms27156960 (PMC13466389; doi:10.3390/ijms27156960)
Supplement: Supplementary file 1 [file ijms-27-06960-s001.zip › ijms-4330194-supplementary.pdf]

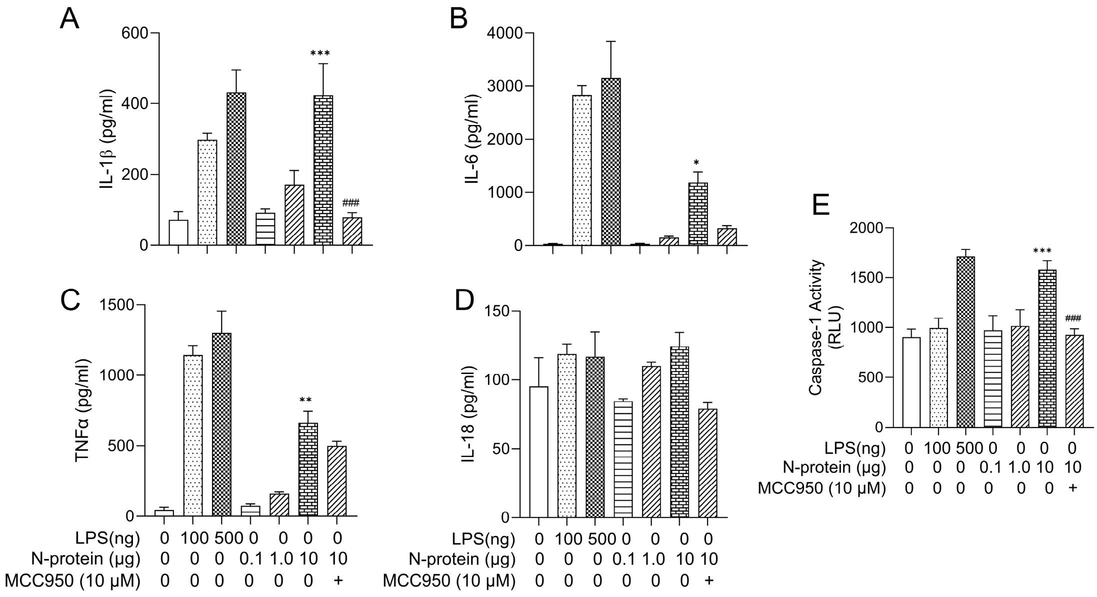

Figure S1. SARS-CoV-2 N-protein promotes cytokine production and caspase-1 activity: Primary rat neonatal microglia treated with SARS-CoV-2 N-protein (0.1, 1.0, and 10.0 μg/ml) showed a dose-response in released cytokines in the collected culture supernatants (A, B, C, and D for IL-1β, IL-6, TNF-α, and IL-18, respectively). Microglial cultures were treated with lipopolysaccharide (LPS) at concentrations of 100 ng/ml and 500 ng/ml for the control. For caspase-1 activity, the collected supernatants were measured for RLU using the Caspase-1 glow kit (E). Before treatment, MCC950 (an NLRP3-specific inhibitor) was used to establish whether cytokine secretion is mediated by inflammasome activation. Microglia were pretreated with the specified inhibitor at the specified concentration for 2 hours before N-protein treatment. * $p < 0.05$, ** $p < 0.01$, *** $p < 0.001$ vs. untreated control and ### $p < 0.001$ vs. SARS-CoV-2 N-protein (10 μg/ml) treatment.

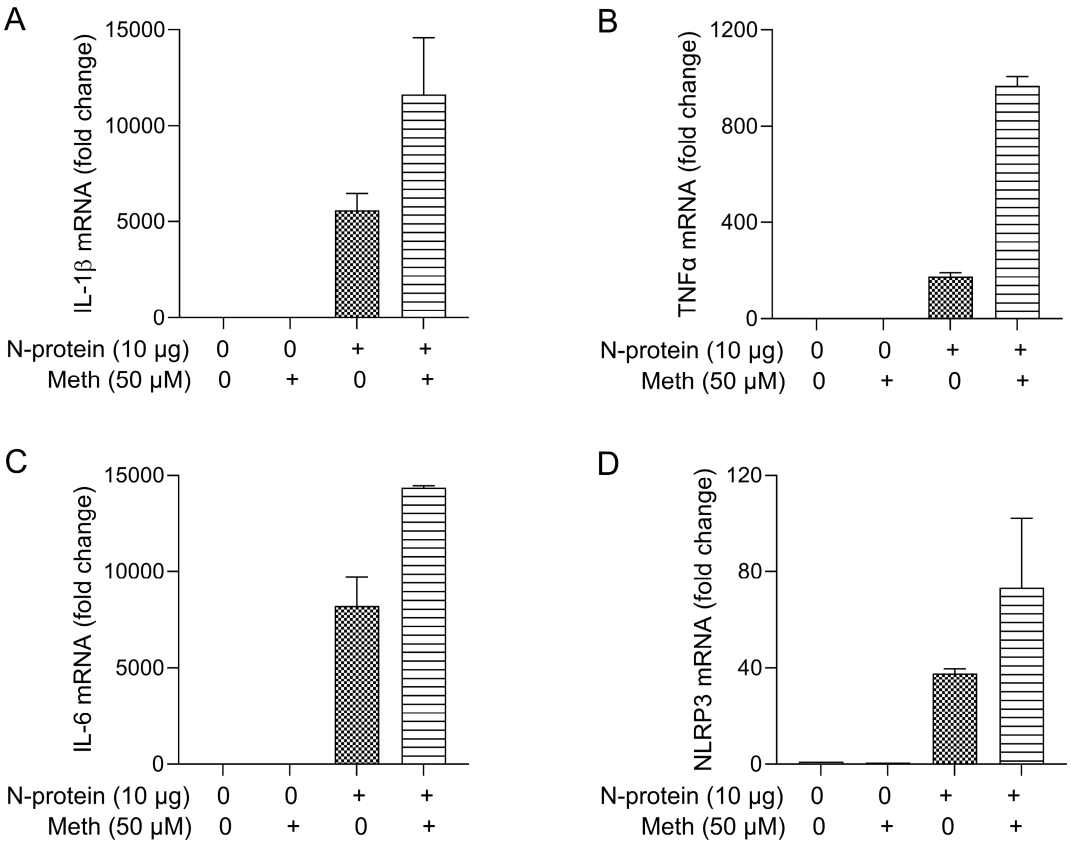

Figure S2. Effect of SARS-CoV-2 N-protein and meth on cytokine expression: Primary rat neonatal microglia were treated with SARS-CoV-2 N-protein (10.0 µg/ml) and meth (50 µg), showing mRNA expression of cytokines and NLRP3, which was measured by qRT-PCR (A, B, C, and D for IL-1β, TNF-α, IL-6, and NLRP3, respectively).

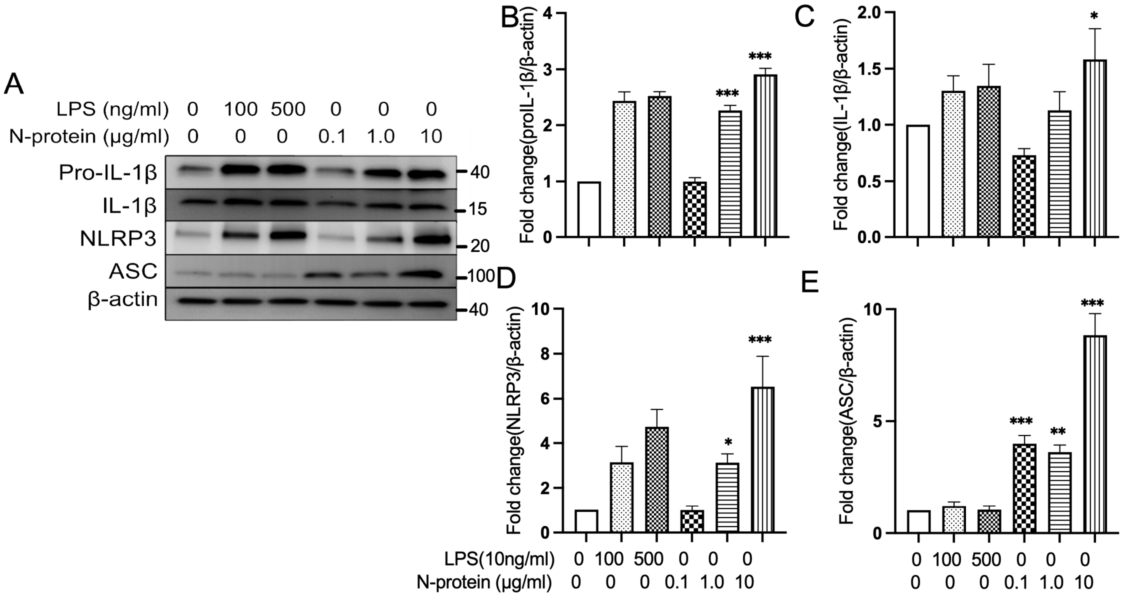

Figure S3. SARS-CoV-2 N-protein promotes expression of inflammasome components. The processing of IL-1β, NLRP3, and ASC in N-protein-treated rat microglial cultures was detected by western blot analysis (A). After

standardization to β-actin, band densitometry was quantified, as shown in the bar graphs for pro-IL-1β (B), IL-1β (C), NLRP3 (D), and ASC (E). * $p < 0.05$, ** $p < 0.01$, *** $p < 0.001$ vs. untreated control.

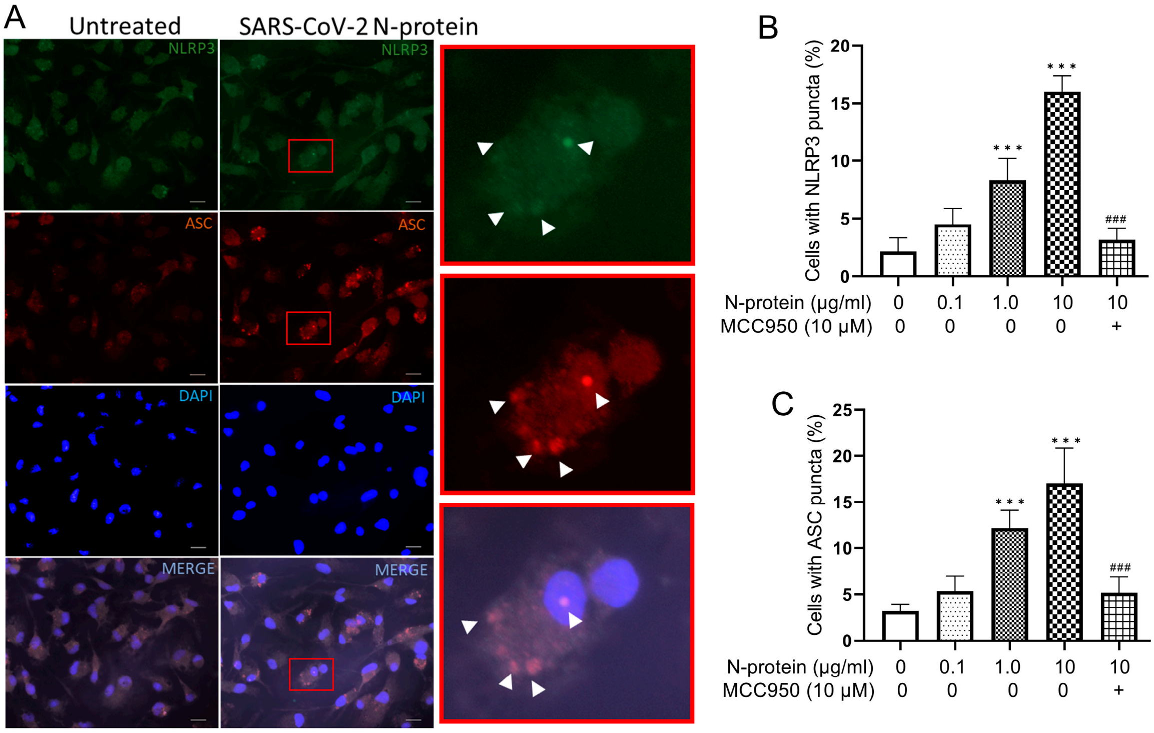

Figure S4. SARS-CoV-2 N-protein-induced NLRP3 and ASC puncta formation, a hallmark of NLRP3 inflammasome activation. The NLRP3 inflammasome activation by the SARS-CoV-2 N-protein was confirmed by quantifying NLRP3 and ASC puncta using immunofluorescence microscopy. Rat neonatal microglial cells treated with N-protein were stained with NLRP3 and ASC-specific antibodies to monitor microglial NLRP3 activation. (A) IFA image after SARS-CoV-2 N-protein treatment. (B) and (C) are bar graphs showing the quantification results of NLRP3 and ASC puncta, respectively. To validate inflammasome activation by the SARS-CoV-2 N-protein, the NLRP3-specific inhibitor MCC950 was administered prior to treatment. *** $p < 0.001$ vs. untreated control and ### $p < 0.001$ vs. SARS-CoV-2 N-protein (10 μg/ml) treatment.

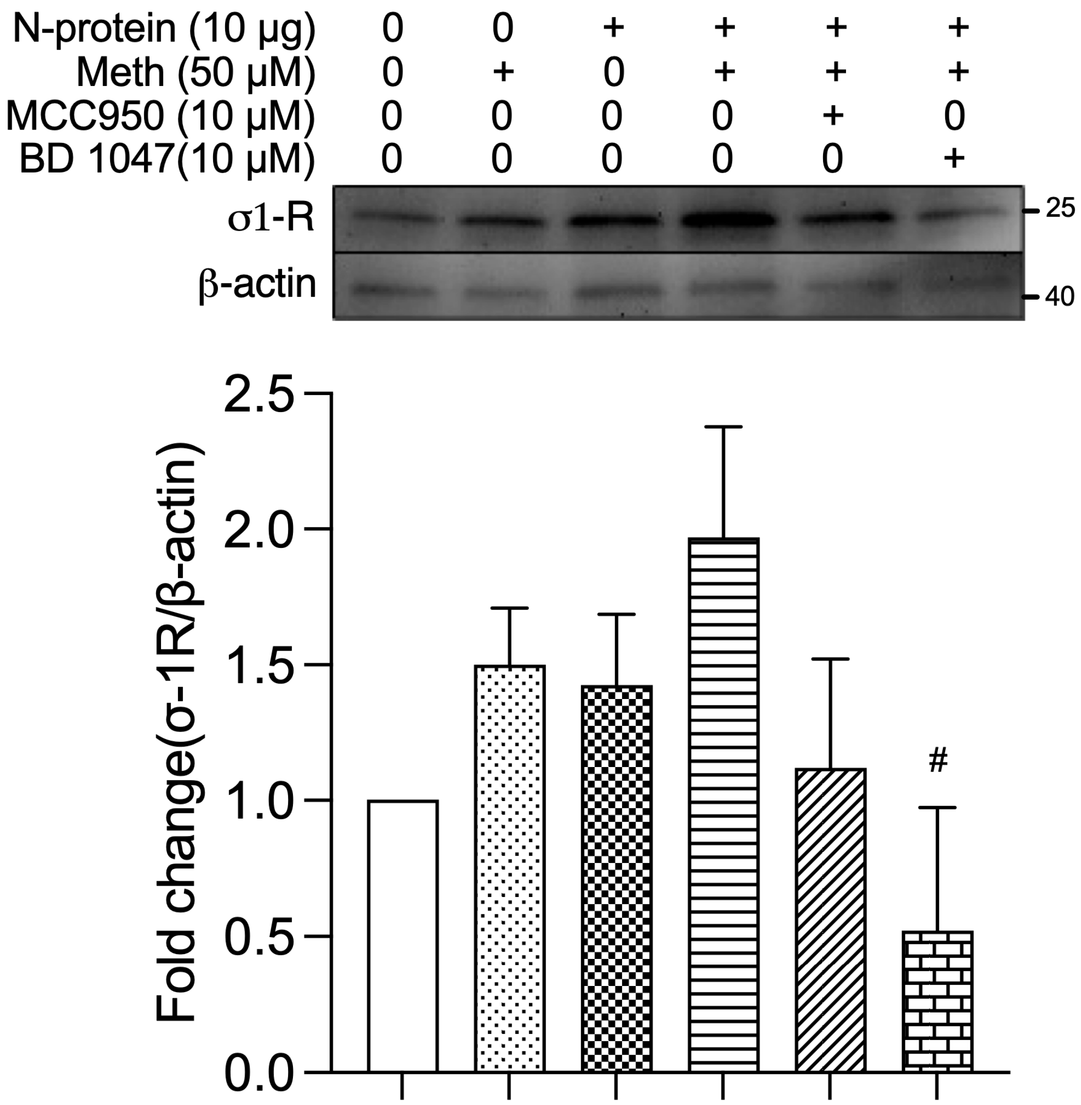

Figure S5. Sigma-1 receptor in meth exacerbation of SARS-CoV-2 N-protein-induced microglial NLRP3 inflammasome activation. To establish the role of the sigma-1 receptor (σ1-R) in SARS-CoV-2 N-protein and meth exacerbation of NLRP3 inflammasome activation, we first measured σ1-R expression by western blot. Rat neonatal microglial cultures were subjected to N-protein treatment for 24 hrs and meth for another 24 hrs. Microglial cultures were pretreated with MCC950 (10 μM), an NLRP3-specific inhibitor, and BD 1047 (10μM), a σ1-R-specific inhibitor, two hrs before N-protein treatment. Cell lysates were subjected to western blotting, and after normalization to β-actin, band intensities were analyzed and quantified as shown in the bar graphs. $^{\#}$ $p < 0.05$ vs. SARS-CoV-2 N-protein (10 μg/ml) and meth (50 μM) treatment.

Table S1. List of primers used in the study.

Primer name	Oligonucleotide sequence
NLRP3 SENSE RAT	TGGTCAAGGAGCATCCAAGCA
NLRP3 ANTISENSE RAT	AAGTGTTCATCCTCAGGCTCAAA
Il1β FORWARD	GACCTGTTCTTTGAGGCTGACA
IL1β REVERSE	AGTCAAGGGCTTGGAAGCAA
TNFα FORWARD	CGCCACCGGCAAGGA
TNFα REVERSE	GACATTCCGGGATCCAGTGA
IL6 SENSE	GAGGAGACTTCACAGAGGAT
IL6 ANTISENSE	TCCTTAGCCACTCCTTCTGT
GAPDH RAT SENSE	GCTGGTCATCAACGGGAAA
GAPDH RAT ANTISENSE	ACGCCAGTAGACTCCACGACA
